# Effect of vitamin C on adrenal suppression following etomidate for rapid sequence induction in trauma patients: a randomized clinical trial

**DOI:** 10.1186/s12871-023-02065-5

**Published:** 2023-04-01

**Authors:** Jafar Rahimi Panahi, Seyed Pouya Paknezhad, Amir vahedi, Kavous Shahsavarinia, Manuchehr Ruhi Laleh, Hassan Soleimanpour

**Affiliations:** 1 Department of Anesthesiology, Faculty of Medicine, Tabriz University of Medical Sciences, Tabriz, Iran; 2 Emergency and trauma care research center, Tabriz University of Medical Sciences, Tabriz, Iran; 3 Department of Pathology, School of Medicine, Tabriz University of Medical Sciences, Tabriz, Iran; 4 Road Traffic Injury Research Center, Tabriz University of Medical Sciences, Tabriz, Iran; 5 Student Research Committee, Tabriz University of Medical Sciences, Tabriz, Iran

**Keywords:** Etomidate, Vitamin C, Rapid sequence intubation

## Abstract

**Background:**

Etomidate is an imidazole derivative that is widely used in the emergency department for Rapid Sequence Intubation (RSI). Although it has a safe hemodynamic profile, there are some concerns about its suppressant effects on the adreno-cortical axis. Vitamin C, as an antioxidant, can play a protective role in this issue.

**Method:**

In a controlled clinical trial, we studied adult traumatic patients who needed RSI with etomidate. In one group underwent RSI with etomidate and cortisol levels were measured three hours later. In the other group, we administered one gram of vitamin C before etomidate administration, and the cortisol level was measured three hours later.

**Results:**

Fifty-one patients have been studied. The serum cortisol level was significantly lower after RSI with etomidate in both groups. In the Vitamin C group, there was a significantly higher cortisol level after RSI in comparison to the control group.

**Conclusion:**

Etomidate can suppress the cortisol level in trauma patients who undergo RSI. Vitamin C can reduce this suppressant effect of etomidate.

**Trial registration:**

IRCT registration number: IRCT20090923002496N11, URL of trial registry record: https://en.irct.ir/trial/34586, Date of trial registration: 19/04/2019. Full date of the first registration: 30/05/2019.

## Introduction

Rapid sequence induction/intubation (RSI) is the standard technique for airway management and intubation in the emergency department (ED). This technique can reduce the risk of aspiration and intubation failure by using an anesthetic agent and a neuro-muscular blocking agent [1, 2]. Using a safe and effective induction agent can maximize intubation success and reduce the risk of adverse effects. Etomidate, an imidazole derivative, is widely used as an induction agent for RSI in EDs. This medication was chosen as a treatment for RSI due to its quick onset of action, safe hemodynamic profile, minimal histamine production, and modest influence on respiratory depression [3–5]. According to certain research, etomidate inhibits the enzyme 11-beta-hydroxylase, which results in adrenal insufficiency [5, 6]. Cortisol, the most abundant endogenous glucocorticoid, can produce less due to this dose-dependent inhibition of 11-beta-hydroxylase [7]. Although some studies in elective surgical patients did not find this suppression to be a significant issue and the conclusions were controversial, others discovered that it may result in traumatic patients needing longer ventilatory support, spending more time in the intensive care unit (ICU), and having a higher risk of developing acquired respiratory distress syndrome (ARDS) [6, 8, 9]. An antioxidant agent is vitamin C. It indicated a little improvement in patients’ prognoses after head trauma. It also protects against inflammation in hemorrhagic shock by activating the enzyme Hemoxigenase-1 [9–11]. Years ago, Boidin et al. introduced vitamin C as a selective treatment for adrenal suppression caused by etomidate. Since then, this has been a debated issue, and the role of vitamin C in the inhibition of adrenal suppression and its clinical significance remains controversial [12]. We conducted a clinical trial to investigate the effect of vitamin C on cortisol levels in trauma patients who underwent RSI with etomidate.

## Material and method

Imam Reza General Hospital, a trauma referral hospital in Tabriz, East Azarbaijan Province, North West Iran, hosted this clinical trial.

**Fig. 1:**
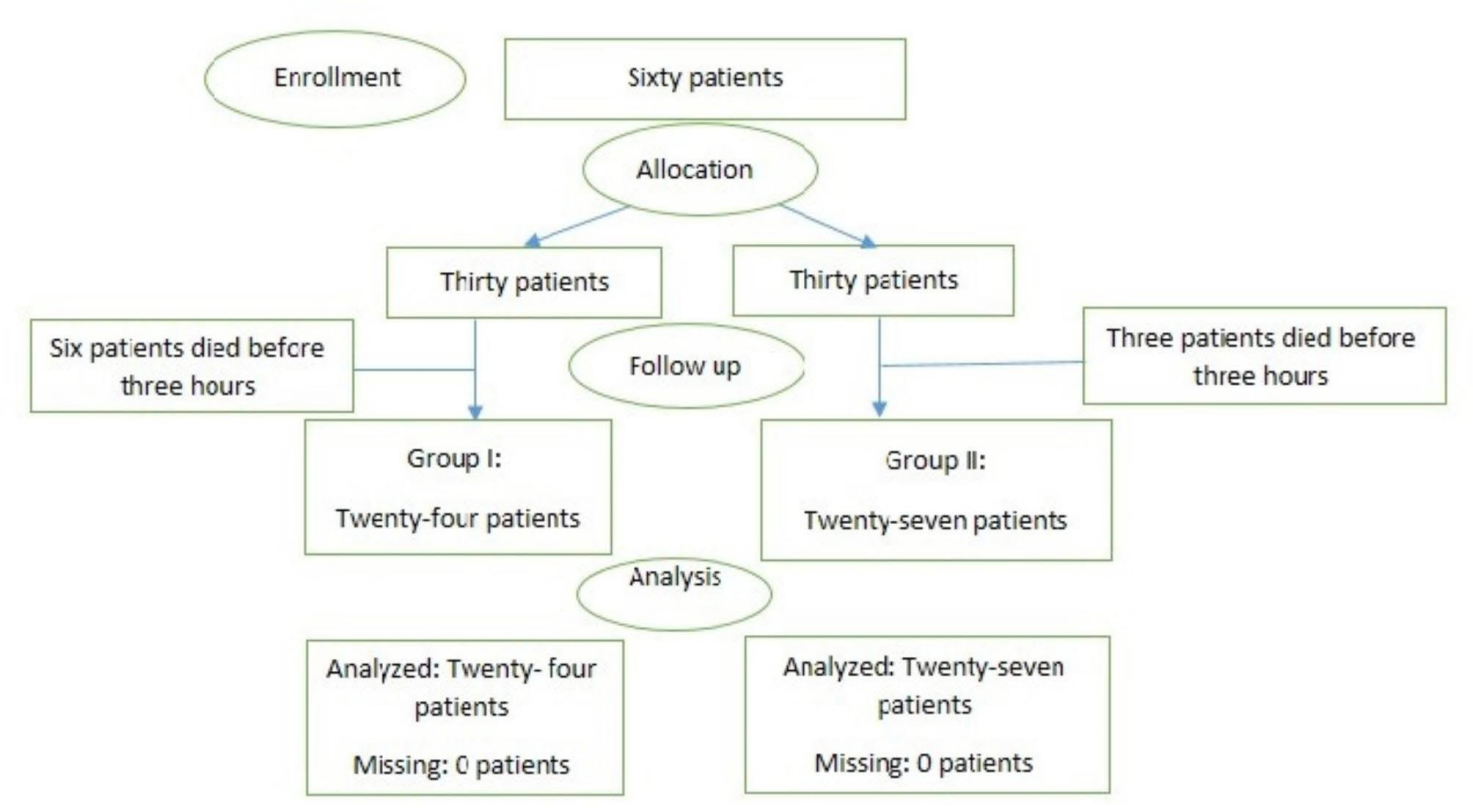
Flow chart of the study

Based on our center admission rate, we estimated that we would have 60 eligible patients to enroll in this trial in a six-month period of time (one eligible patient every three days). Patients with a history of trauma who needed airway management with RSI were divided into two groups randomly. Totally, sixty patients entered the study. Randomization was done by using a random number table. Patients under the age of 18, pregnant women, people with a history of cardiac, renal, or endocrine disorders, and people who have taken corticosteroid medication in the past were all excluded. In all patients, a blood sample for serum cortisol level was obtained prior to RSI. In group I, patients underwent RSI with 0.3 mg/kg Etomidate (Aburaihan Pharmaceutical Co., Iran) and 1.5 mg/kg succinylcholine (Chandra Bhagat Pharma Limited, India), and three hours later, serum cortisol levels were checked in these patients. As a placebo, these patients received 10 CC of normal saline (two shots of five CC of saline) intravenously. In group II, one-gram (500 mg/5 cc in two boluses) of intravenous vitamin C (Sobhan Darou Pharmaceutical Co., Iran) was injected in the pretreatment step of RSI, and then patients underwent RSI with Etomidate and succinylcholine. Serum cortisol levels were checked three hours later. All patients received fentanyl (1 micg/kg) and midazolam (0.02 mg/kg) for pretreatment. Patient recruitment began on May 30, 2019. Figure 1 shows the consortium flowchart of the study.

The data was analyzed using SPSS Ver 22 software. The results were reported as frequency (percentage) and median (interquartile range). To determine the normality of data distribution, the Kolmogorov-Smirnov test was used. To analyze the data, other methods of inferential statistics were used, such as the Chi-square test, Mann-Whitney U test, and Wilcoxon signed rank (due to the non-normal nature of the data). P < 0.05 was considered as the level of statistical significance. This study was approved by the ethical committee of Tabriz University of Medical Sciences under the number IR.TBZMED.REC.1397.925. Protocol of this clinical trial was submitted in Iranian registry of clinical trials under the ID of IRCT20090923002496N11.

## Results

Sixty patients entered the study in two groups of 30 each. Patients who died within the first three hours were excluded. Finally, fifty-one patients enrolled in the trial. There were 24 patients in group I and 27 patients in group II. In group I, 15 patients were male and nine were female. In group II, 17 patients were male and 10 were female. There was no statistical difference in gender distribution between the two groups.

**Fig. 2:**
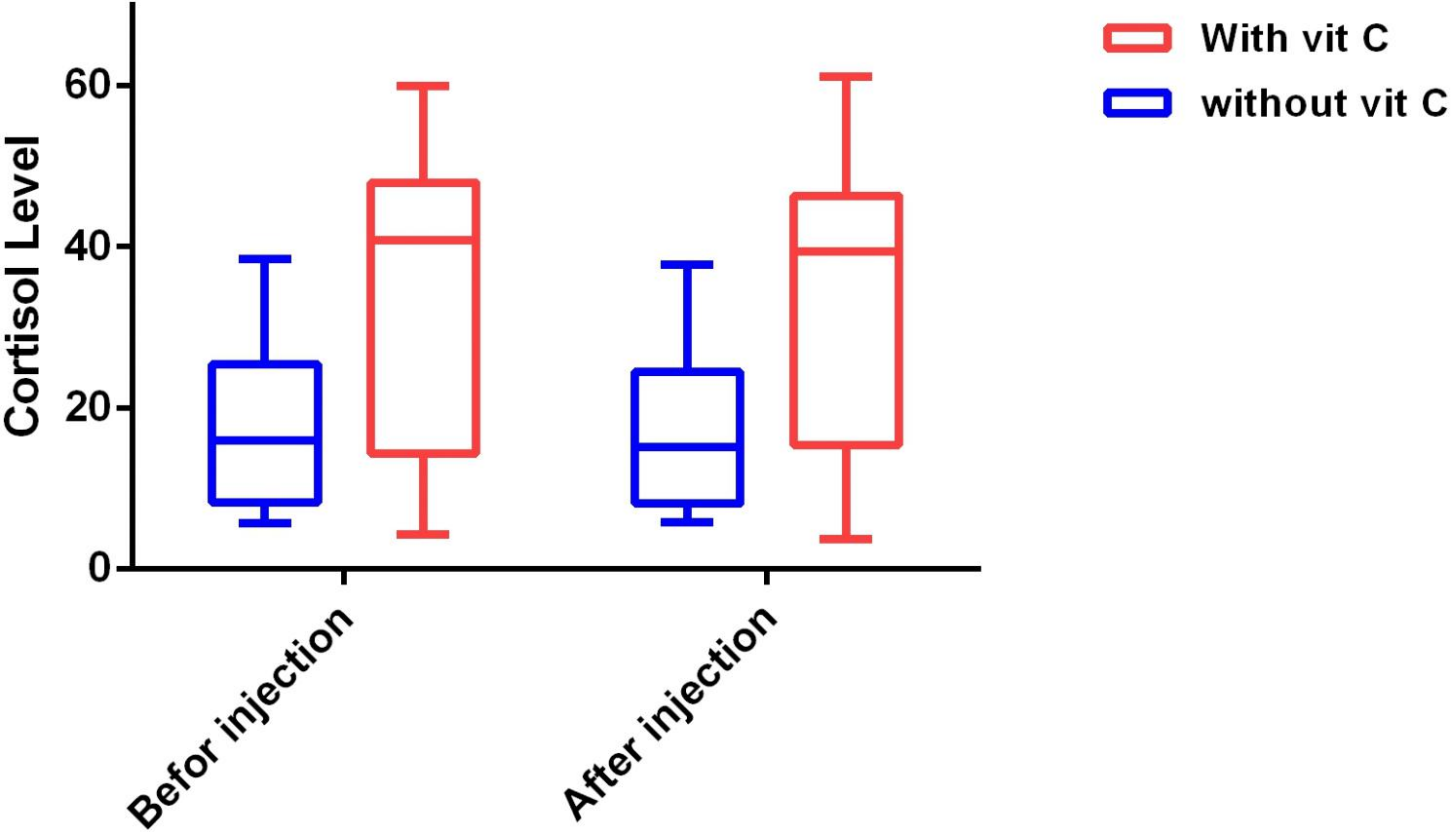
Cortisol level changes in both groups (Before and after etomidate injection)

The average age of patients in group I was 64.5 years, with the lowest age being 18 years and the highest being 89 years. The average age of patients in Group II was 65 years, with the lowest age being 18 years and the highest being 86 years. There was no statistically significant difference in terms of age between the two groups (P = 0.770) (Table 1). The median serum cortisol level of patients in Group I before induction, was 15.9 (with the lowest level of 5.7 and the highest level of 38.4). The median serum cortisol level of patients in Group II before induction was 40.8 (with the lowest level being 4.3 and the highest level being 59.9). A Statistically significant difference was observed in terms of serum cortisol level before induction between the two groups (P = 0.002) (Fig. 2). The median serum cortisol level of patients in Group I, measured three hours after induction, was 15.05, with the lowest level being 5.8 and the highest level being 37.8. The median serum cortisol level of patients in group II, measured three hours after induction, was 39.4, with the lowest level being 3.7 and the highest level being 61.1. A statistically significant difference was observed in terms of serum cortisol levels after injection between the two groups (P < 0.001). A statistically significant difference (P = 0.005) was found in the intra-group comparison of the serum cortisol levels of patients in the group receiving etomidate without vitamin C injection (measured after injection compared to measurement before injection). No statistically significant difference was found (P = 0.564) in the intra-group comparison of the serum cortisol levels of the patients receiving etomidate and vitamin C injection (measured after injection compared to measurement before injection). (Table 2).

**Table 1:** Demographic characteristics of patients

Variable		Group		P - Value
		Without Vit C n = 24 Frequency (percent)	With Vit C n = 27 Frequency (percent)	
**Sex**	Male	15 (62.5)	17 (63)	0.973
	Female	9 (37.5)	10 (37)	
**age**		64.5 (73.75–40.25)^€^	65 (77–46) ^€^	0.770

^€^Median (Interquartile)

**Table 2:** Comparison of Cortisol levels between two groups, before and after etomidate injection

		Group		P - Value
		Without Vit C n = 24 Median (Interquartile)	With Vit C n = 27 Median (Interquartile)	
**Cortisol level**	Before injection	15.9 (25.37–8.17)	40.8 (47.9–14.3)	**0.002**
	After injection	15.05 (24.42–8.1)	39.4 (46.3–15.4)	**<0.001**
	**P - Value**	**0.005**	**0.564**	

## Discussion

Our investigation demonstrates that there is a statistically significant difference between the two groups’ serum cortisol levels prior to and following the administration of etomidate. In the patients who received Vitamin C, the amount of serum cortisol following etomidate injection did not fall as significantly than in the patients who did not. Etomidate has the potential to suppress the adrenal response to significant stress following trauma, which could result in life-threatening adverse effects in trauma patients. Charuvi et al. found that a single dose of etomidate in patients undergoing laparoscopic cholecystectomy can cause a suppression in serum cortisol levels [6]. The results of our study are consistent with these findings. According to Warner et al., patients with multiple trauma who underwent RSI with etomidate had a higher rate of ARDS [9]. In our study and Charuvi’s study, in contrast to Warner’s study, no complications related to cortisol suppression were found. A study showed that a vitamin C injection given 20 min prior to etomidate administration in rabbits significantly increased the serum cortisol level and prevented the inhibitory effect of etomidate on the adrenocortical axis [13]. In human studies, vitamin C administration before cardiopulmonary bypass in cardiac surgery could prevent adrenocortical axis inhibition [14]. Our findings are in line with the research done by Nooraei et al., who looked at 40 individuals who had elective laparotomies and underwent RSI with etomidate. Twenty individuals received vitamin C prior to receiving an injection of etomidate, while the other 20 received merely saline. In contrast to the vitamin C-treated group, the control group in their study exhibited a decrease in cortisol levels. Additionally, the control group’s C-reactive protein (CRP) level increased, but not in individuals receiving vitamin C. [15]. Deepanwita Das et al. investigated seventy patients who underwent elective heart surgery. Patients who took one gram of vitamin C orally every day for seven days prior to surgery had greater cortisol levels than the control group, which is consistent with our study. The decrease in serum cortisol level was not only observed in the first hour after RSI but also for 24 h after induction with etomidate [14]. Contrary to the results of our study, Nathan et al. did not find vitamin C to be a protective agent against adrenal suppression who studied a small group of patients (16 patients in two groups) and found a lower cortisol level in patients receiving vitamin C as premedication for RSI [16].

### Limitation

This was a single center study with a limited sample size. A larger sample size in a multicenter study could improve the power of the study. We did not study the effect of cortisol levels on the outcomes of patients and did not follow the final outcomes of patients in two groups. Based on the study protocol and method of randomization, we could not match our patients in terms of admission time. The cortisol serum level has a circadian cycle, and a difference in admission time may cause significant differences in the serum cortisol level. This caused a statistically significant difference in the baseline serum cortisol level between two groups in our study.

## Conclusion

According to the majority of research done as well as our investigation, it is evident that injecting vitamin C prior to injecting etomidate in the induction stage of intubation can avoid adrenal inhibition and reduce the amount of serum cortisol level suppression caused by etomidate. Although some studies have recommended alternative medications such as midazolam in place of etomidate, this medication is still the first choice for unstable trauma patients due to the minimal adverse effects of etomidate.

However, due to the numerous side effects of corticosteroids, more emphasis is placed on vitamin C, which, in addition to its antioxidant effects, can prevent adrenal inhibition by etomidate.

## Data Availability

The datasets generated during and analysed during the current study are not publicly available due to restriction of ethic committee of Tabriz University of Medical Sciences but are available from the corresponding author on reasonable request.

## Abbreviations

ED: Emergency Department
RSI: Rapid Sequence Intubation
ARDS: Acquired Respiratory Distress Syndrome
