# Correction: Effect of vitamin C on adrenal suppression following etomidate for rapid sequence induction in trauma patients: a randomized clinical trial

**DOI:** 10.1186/s12871-023-02090-4

**Published:** 2023-05-11

**Authors:** Jafar Rahimi Panahi, Seyed Pouya Paknezhad, Amir Vahedi, Kavous Shahsavarinia, Manuchehr Ruhi Laleh, Hassan Soleimanpour

**Affiliations:** 1 Department of Anesthesiology, Faculty of Medicine, Tabriz University of Medical Sciences, Tabriz, Iran; 2 Emergency and trauma care research center, Tabriz University of Medical Sciences, Tabriz, Iran; 3 Department of Pathology, School of Medicine, Tabriz University of Medical Sciences, Tabriz, Iran; 4 Road Traffic Injury Research Center, Tabriz University of Medical Sciences, Tabriz, Iran; 5 Student Research Committee, Tabriz University of Medical Sciences, Tabriz, Iran

**Correction: BMC Anesthesiol 23, 104 (2023)**

**https://doi.org/10.1186/s12871-023-02065-5**

Following publication of the original article [1], the authors reported an error in the author group. The family name of Kavous Shahsavarinia was spelt incorrectly.

The incorrect author name is: Kavous Shasavainia.

The correct author name is: Kavous Shahsavarinia.

The affiliations of the authors Kavous Shahsavarinia and Hassan Soleimanpour were not captured correctly. Kavous Shahsavarinia should be affiliated to *Road Traffic Injury Research Center, Tabriz University of Medical Sciences, Tabriz, Iran* and author Hassan Soleimanpour should be affiliated to *Emergency and trauma care research center, Tabriz University of Medical Sciences, Tabriz, Iran*.

The author group has been updated above and the original article [1] has been corrected.
